# Long-Term Trends in Human Parainfluenza Virus Types 1, 2, and 3 Infection in Korea (2007–2024)

**DOI:** 10.3390/pathogens14111159

**Published:** 2025-11-14

**Authors:** Yu Jeong Kim, Jeong Su Han, Jae-Sik Jeon, Sung Hun Jang, Qianwen Wang, Jae Kyung Kim

**Affiliations:** 1 Department of Biomedical Laboratory Science, College of Health Sciences, Dankook University, Cheonan-si 31116, Republic of Korea; bkh04117@naver.com (Y.J.K.); jshan1162@naver.com (J.S.H.); zenty87@naver.com (J.-S.J.); 2 Department of Medical Laser, Graduate School of Medicine, Dankook University, Cheonan-si 31116, Republic of Korea; well8143@naver.com; 3 Department of Public Health and Health, Suzhou Vocational Health College, Suzhou 215009, China; wangqianwenins@163.com

**Keywords:** COVID-19 pandemic, human parainfluenza virus (HPIV), seasonality, sustainable development goals (SDG 3: good health and well-being)

## Abstract

This study investigated the long-term trends in human parainfluenza virus (HPIV) types 1, 2, and 3 in Korea by year, age group, and season. A total of 23,284 nasopharyngeal swabs collected from patients with respiratory symptoms at a tertiary hospital in Korea between 2007 and 2024 were tested for HPIV using real-time reverse-transcription polymerase chain reaction. Of the 23,284 specimens tested, 481 were positive for HPIV-1, 164 for HPIV-2, and 1102 for HPIV-3. HPIV-3 showed the highest incidence between 2010 and 2016, a decline after 2018, a sharp decline during the 2020 COVID-19 pandemic, and a resurgence in 2021. HPIV-1 and HPIV-2 incidence fluctuated between 2007 and 2019, followed by a sharp decline in 2020. HPIV-3 activity peaked in spring and summer, whereas HPIV-1 and HPIV-2 peaked in autumn. For all three types, infection rates were generally highest among children aged 1–12 years, followed by those in infants, but infection rates varied significantly by type, year, season, and age group. These findings emphasize targeted pediatric prevention, predictive modeling of seasonal peaks, and continued molecular surveillance to clarify the genetic and antigenic diversity of HPIV types after the pandemic, supporting the Sustainable Development Goals (SDG 3 for Good Health and Well-Being).

## 1. Introduction

Human parainfluenza virus (HPIV) belongs to the family *Paramyxoviridae* [1,2]. It is an enveloped, non-segmented, single-stranded negative-sense RNA virus, with a genome length of 14.9–17.3 kb. The genome encodes six structural proteins: nucleocapsid (N), phosphoprotein (P), matrix, fusion (F), hemagglutinin-neuraminidase (HN), and large (L) proteins. Among these, the F and HN glycoproteins serve as membrane-binding proteins, while the N, P, and L proteins are involved in nucleocapsid formation [3].

HPIV is classified into four types: HPIV-1, HPIV-2, HPIV-3, and HPIV-4, each with distinct seasonal patterns and clinical characteristics [4]. HPIV-1 and HPIV-2 are commonly associated with croup, whereas HPIV-3 and HPIV-4 are more frequently associated with bronchitis and pneumonia [5]. HPIV is an important cause of respiratory infection, especially in children, and can lead to severe diseases, such as laryngitis, bronchitis, and pneumonia [6]. HPIV infection occurs primarily in children, and research on the epidemiological patterns of HPIV in adolescents and adults is limited [7,8]. Despite ongoing efforts to develop a vaccine, no commercial vaccines are currently available owing to the diversity of virus types, suboptimal immune responses, and safety concerns in young children [9,10]. Many previous epidemiological studies of HPIV have been limited by a short surveillance period, narrow age-group focus, or analysis of a single virus type. Moreover, few studies have assessed infection trends spanning the COVID-19 pandemic period [11]. The COVID-19 pandemic substantially disrupted the circulation of seasonal respiratory viruses and led to shifts in age distribution and seasonal dynamics.

Accordingly, this study aimed to characterize the long-term epidemiological patterns and temporal trends of HPIV types 1, 2, and 3 infection in Korea from 2007 to 2024, by year, season, and age group, and to provide evidence to inform tailored prevention strategies, guide vaccination priorities, and strengthen infectious disease surveillance. HPIV-4 was not included in this study because the RT-PCR diagnostic assay used at the study hospital does not target this type.

## 2. Materials and Methods

In this study, we analyzed the annual, seasonal, and age-specific infection patterns of HPIV-1, HPIV-2, and HPIV-3 using real-time reverse-transcription polymerase chain reaction (RT-PCR) data collected at Dankook University Hospital in Cheonan, Korea, from 2007 to 2024. This study was approved by the Institutional Review Board of Dankook University (approval number: DKU 2025-02-004-003). All data were collected and managed in accordance with the principles of the Declaration of Helsinki, and only fully anonymized data without personally identifiable information were analyzed. Given the retrospective nature of the study using anonymized data, the requirement for informed consent was waived.

### 2.1. Data Collection

Testing was performed on inpatients and outpatients presenting with influenza-like symptoms such as fever, cough, and sore throat, or respiratory infection. The decision to test patients for HPIV was at the discretion of the attending clinician. The dataset included test dates, test results, and patient demographics (age and sex) of all specimens tested for HPIV at the hospital during the study period.

### 2.2. Data Processing

The data underwent preprocessing to ensure suitability for analysis. Records with missing information on age, sex, sampling date (year and month), or test results were excluded by the data provider during preprocessing; therefore, information on the number of samples with missing or invalid results was not available. Age was categorized according to the World Health Organization classification into the following groups: infants (0 years), children (1–12 years), adolescents (13–18 years), adults (19–64 years), and older adults (≥65 years). Season was classified as follows: spring (March to May), summer (June to August), autumn (September to November), and winter (December to February).

### 2.3. RNA Extraction and Real-Time PCR

Nasopharyngeal swab specimens were either tested immediately after collection or stored at 4 °C and tested within 24 h. Viral RNA was extracted from the nasopharyngeal swabs using a commercial kit (QIAamp Viral RNA Mini Kit, Qiagen, Germany). The RNA specifically bound to the QIAamp silica membrane and was eluted with the buffer provided in the kit. All procedures were performed according to the manufacturer’s instructions. The extracted RNA was analyzed using a Respiratory Virus RT-PCR kit (LG Life Sciences, Seoul, Republic of Korea). HPIV genes were detected with virus-specific TaqMan probes along with the primers and probes supplied in the kit. For HPIV-1, HPIV-2, and HPIV-3, the HN gene was targeted to allow differentiation of each type. PCR amplification was conducted using the SLAN RT-PCR System (LG Life Sciences, Seoul, Republic of Korea), and the results were interpreted in accordance with the manufacturer’s protocol.

### 2.4. Data Analysis

Data analysis was performed using R software (version 4.5.1). The primary outcome was HPIV positivity. As the variables analyzed in this study, including HPIV positivity, calendar year, age group, and season, were all categorical, chi-square tests were used to compare groups. To assess the effect of the COVID-19 pandemic, we compared HPIV positivity during the pre-pandemic (2017–2019), pandemic (2020), and post-pandemic (2021–2024) periods using chi-square tests. In addition, pairwise Fisher’s exact *p*-values (Benjamini–Hochberg-adjusted) were calculated as a supplementary analysis owing to small cell counts. Statistical significance was set at *p* < 0.05. Use of Artificial Intelligence Tools: During the preparation of this manuscript, the authors used ChatGPT-5 (OpenAI, San Francisco, CA, USA) solely for English language refinement and style improvement. The authors thoroughly reviewed and verified all AI-assisted text to ensure accuracy, consistency, and scientific integrity. No AI tools were used for data analysis, interpretation, or generation of scientific content.

## 3. Results

### 3.1. Study Population

Table 1 shows the demographic distribution of patients who were tested for HPIV and reflects the demographic composition of the population tested.

### 3.2. Annual Incidence

**Figure 1:**
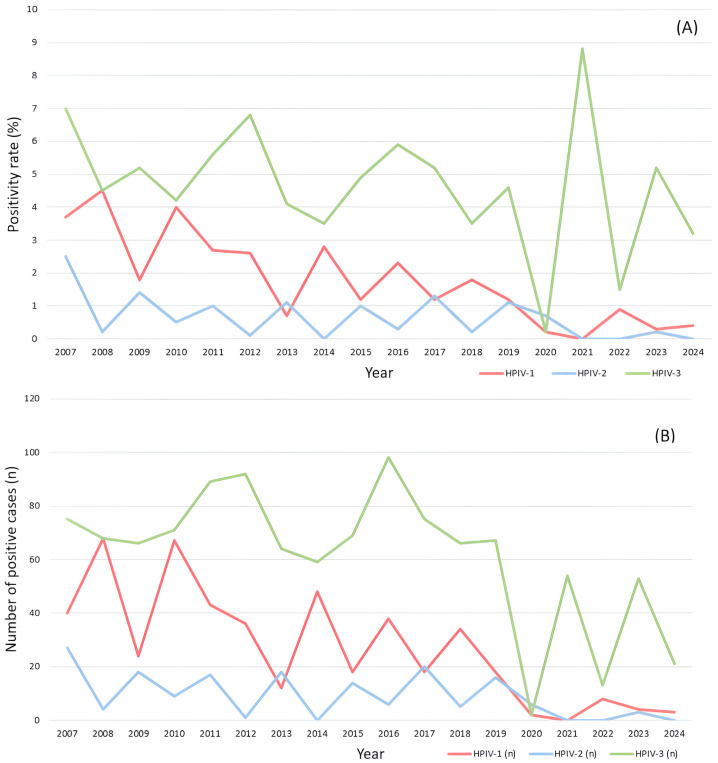
Annual positivity rate and absolute case counts of human parainfluenza virus (HPIV) types 1–3 detected in nasopharyngeal specimens from patients with respiratory symptoms at a tertiary hospital in Korea from 2007 to 2024. (**A**) Annual positivity rates (%) of HPIV-1, HPIV-2, and HPIV-3 as a proportion of samples tested. (**B**) Annual number of laboratory-confirmed positive cases (n) for each HPIV type during the same period. HPIV-1: red line; HPIV-2: blue line; and HPIV-3: green line.

Of the 23,284 specimens tested, 481 were positive for HPIV-1, 164 for HPIV-2, and 1102 for HPIV-3 (Table S1). Over the 18-year surveillance period, annual positivity rates ranged from 0 to 4.5% for HPIV-1, 0 to 2.5% for HPIV-2, and 0.2 to 8.8% for HPIV-3. Between 2010 and 2016, the positivity rates were consistently higher for HPIV-3 (4.2–6.8%) than for the other two types. Between 2014 and 2017, HPIV-3 ranged between 3.5% and 5.2%. HPIV-3 incidence gradually declined in 2019. With the onset of the COVID-19 pandemic in 2020, positivity declined sharply across all types (HPIV-1: 0.2%; HPIV-2: 0.7%; HPIV-3: 0.2%). However, HPIV-3 showed a resurgence in 2021, peaking at 8.8%, before declining to 3.2% in 2024. The annual incidence of HPIV infection differed significantly according to calendar year and virus type (*p* < 0.001; Figure 1A,B).

The positivity rates of all types also differed significantly by period. The HPIV-3 positivity rate decreased from 4.42% (208/4702) in 2017–2019 to 0.25% (2/792) and then rebounded to 4.49% (141/3142) in 2021–2024 (*p* < 0.001). The HPIV-1 positivity rate decreased from 1.49% (70/4702) in 2017–2019 to 0.25% (2/792) in 2020 and remained low (0.48%, 15/3142) in 2021–2024 (*p* < 0.001). The HPIV-2 positivity rate gradually decreased from 0.87% in 2017–2019 to 0.76% in 2020 and remained low (0.10%) in 2021–2024 (*p* < 0.001).

### 3.3. Seasonal Infection Patterns

**Figure 2:**
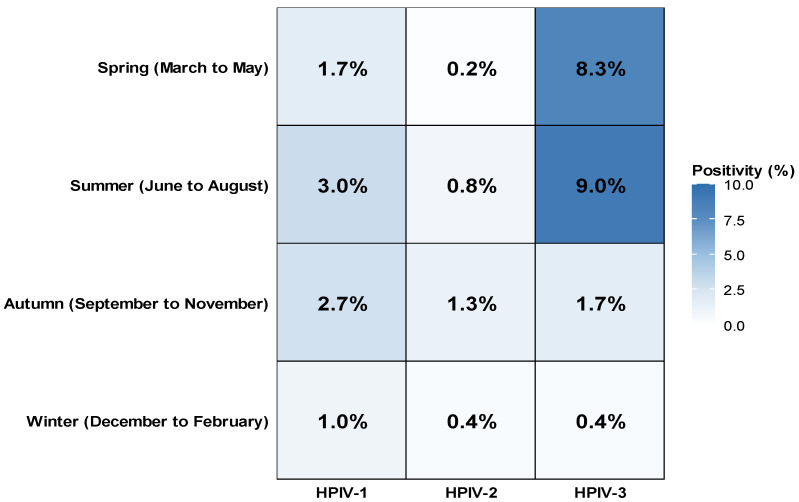
Seasonal distribution of HPIV-1, HPIV-2, and HPIV-3 positivity rates in Korea (2007–2024). The heatmap illustrates the seasonal variation in positivity rates for HPIV-1, HPIV-2, and HPIV-3. The highest activity of HPIV-3 was observed during spring and summer, whereas HPIV-1 and HPIV-2 peaked in autumn. Color intensity corresponds to the positivity rate (%).

HPIV infection was detected throughout all four seasons; however, the seasonality differed by type. The seasonal distribution of HPIV infection is shown in Table S2 and Figure 2. Seasonal variation differed significantly among the three HPIV types (*p* < 0.001). The HPIV-1 positivity rate was highest in summer (3.0%) and autumn (2.7%) and lower in spring (1.7%) and winter (1.0%). The HPIV-2 positivity rate was highest in autumn (1.3%) and summer (0.8%) and lower in winter (0.4%) and spring (0.2%). The HPIV-3 positivity rate was highest in summer (9.0%) and spring (8.3%) and lower in autumn (1.7%) and winter (0.4%).

### 3.4. Infection Patterns by Age Group

**Figure 3:**
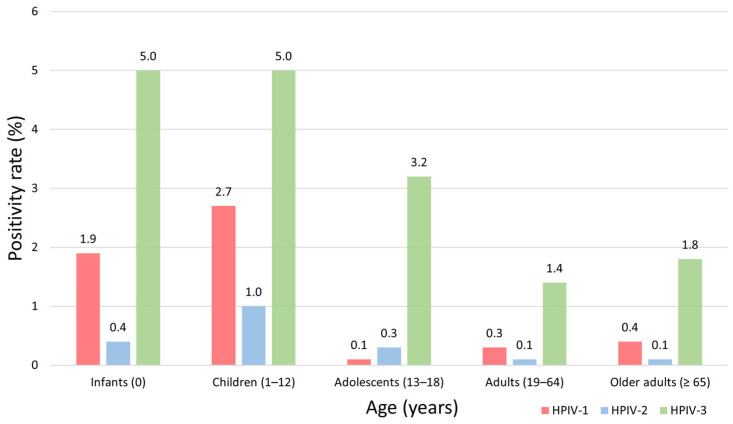
Age-specific positivity rates of human parainfluenza virus (HPIV) types 1–3 in Korea, 2007–2024. The bar chart presents the age-specific positivity rates (%) of HPIV-1, HPIV-2, and HPIV-3 detected in nasopharyngeal specimens collected between 2007 and 2024. The age groups, infants (<1 year), children (1–12 years), adolescents (13–18 years), adults (19–64 years), and older adults (≥65 years) are shown on the *X*-axis, and the positivity rates (%) are shown on the *Y*-axis. Bars are color-coded by virus type: HPIV-1 (red), HPIV-2 (blue), and HPIV-3 (green). The numerical values above each bar indicate the corresponding positivity rate (%) for that age group.

The number of samples tested in each age group was 4543 in infants (<1 year), 10,537 in children (1–12 years), 577 in adolescents (13–18 years), 2935 in adults (19–64 years), and 4692 in older adults (≥65 years). The age-specific distribution by HPIV type is shown in Table 2 and the age-specific positivity rate by HPIV type is shown in Figure 3. Among the three HPIV types combined, the overall positivity rate was highest in children (8.7%), followed by infants (7.3%), adolescents (3.6%), older adults (2.3%), and adults (1.8%) (*p* < 0.001). HPIV-3 consistently exhibited the highest positivity across all age groups, particularly among children (5.0%) and infants (5.0%). HPIV-1 positivity was also higher in children (2.7%) and infants (1.9%) than in the older age groups, whereas HPIV-2 positivity rates were low (≤1.0%) in all age groups.

## 4. Discussion

This study analyzed the epidemiological characteristics of HPIV-1, HPIV-2, and HPIV-3 using data collected between 2007 and 2024. The results confirmed that HPIV infection patterns varied by year, season, and age group. The HPIV positivity rate increased between 2014 and 2017. This may be related to the periodic epidemic nature of the virus or climate changes during this period [9,11]. Although meteorological parameters such as temperature and humidity were not analyzed in this study, previous research conducted in Korea has reported that the circulation of respiratory viruses, including HPIV, is associated with ambient temperature and relative humidity, particularly during the cold winter months [12]. Therefore, the fluctuations observed between 2014 and 2020 may partly reflect climate-related variability in viral transmission. Consistent with previous studies, our findings also demonstrated a marked decline in HPIV detection during the early phase of the COVID-19 pandemic, followed by a resurgence beginning in 2021, particularly for HPIV-3 [9,13]. Previous research has reported a rapid resurgence of HPIV infection after the relaxation of COVID-19 mitigation measures such as mask-wearing and social distancing. National surveillance data from the Korea Influenza and Respiratory Viruses Surveillance System (KINRESS) also demonstrated a marked resurgence of HPIV activity following the relaxation of COVID-19 mitigation measures in 2021, with HPIV-3 being the predominant type [14]. The trends observed in this single-center study are consistent with the national findings, suggesting that the rebound in HPIV circulation after 2021 reflected a nationwide pattern rather than a localized outbreak. Other studies have also shown that children have the highest positivity rates for all HPIV types, followed closely by infants [15], highlighting the need for focused surveillance and preventive strategies in this age group [9,14,15]. A similar decline in incidence during the COVID-19 pandemic, followed by a resurgence, has been reported for other respiratory viruses, including influenza and respiratory syncytial virus, suggesting that public health interventions implemented during the pandemic contributed to suppressing the spread of respiratory viruses [16,17,18].

The overall incidence of HPIV infection was higher in spring and autumn; however, the seasonal pattern differed by HPIV type. HPIV-1 circulated predominantly in summer and autumn, HPIV-2 circulated predominantly in autumn but was relatively distributed across all four seasons, and HPIV-3 circulated predominantly in spring and summer. These seasonal patterns are consistent with those reported in previous studies [19].

The higher infection rates among children than among adolescents and adults [1,20] is probably attributable to their immature immune systems. Although detailed clinical information was not available on the patients in our study, previous reports suggest that HPIV-1 and -2 are strongly associated with croup, whereas HPIV-3 more frequently causes bronchiolitis and pneumonia [21]. Although the incidence of infection was lower in older adults than in other age groups, their increased vulnerability to severe outcomes due to comorbidities and immunosenescence warrants targeted infection control measures for this age group [22]. Age-related differences in susceptibility should be considered when developing prevention strategies.

HPIV vaccine development remains in its early stages. Currently, no HPIV vaccines are commercially available [10,23,24,25,26,27]. The lack of vaccines limits the availability of effective prevention strategies for high-risk groups such as young children and older adults; therefore, HPIV vaccines are urgently needed. Previous studies have reported that two live-attenuated vaccine candidates targeting HPIV-3 demonstrated safety and immunogenicity in Phase 1 clinical trials [22]. Similarly, trials of live-attenuated candidate HPIV-1 and HPIV-2 vaccines showed reduced HPIV replication in adults and seropositive children, but in seronegative children, they exhibited insufficient immunogenicity [23,24].

In the absence of effective vaccines, accurate and timely diagnosis remains critical. The consistent detection of HPIV-3 using RT-PCR demonstrates the utility of molecular assays for differentiating HPIV from other respiratory pathogens with similar clinical presentations. In children’s wards, timely identification of HPIV using molecular assays could reduce unnecessary antibiotic prescriptions and facilitate isolation of infectious patients.

This study has several limitations. First, the data were derived from a single tertiary hospital, which may limit the generalizability of the results. Nevertheless, the long surveillance period and large sample size provide important insights into the long-term circulation patterns of HPIV. Second, clinical information such as underlying comorbidities and disease severity, including hospitalization and intensive care outcomes, was not systematically collected. Furthermore, this study focused exclusively on HPIV detection without evaluating concurrent infection with other respiratory viruses. Because information on coinfection with viruses such as respiratory syncytial virus (RSV), influenza virus, adenovirus, and rhinovirus was not available, their possible contribution to the observed HPIV trends could not be assessed. Future studies integrating HPIV data with data on coinfection with other respiratory viruses are warranted to elucidate potential interactions between different HPIV types and other respiratory viruses. Third, HPIV-4 was not considered in this study because the diagnostic kit that was used did not detect HPIV-4; therefore, the overall burden of HPIV infection may have been underestimated. Because this study was based solely on RT-PCR detection without molecular sequencing, potential genetic variations and lineage shifts among circulating HPIV types could not be evaluated. This limitation may have affected the interpretation of the post-COVID-19 pandemic epidemiological changes. Further studies integrating genomic and antigenic analyses, particularly of the HN and F genes, are needed to clarify the evolutionary mechanisms underlying HPIV re-emergence and seasonal dynamics.

Future studies should adopt multicenter designs to enable regional comparisons and integrate clinical and virological data, as well as include all HPIV types. Such approaches would not only refine the interpretation of epidemiological trends but also clarify the genetic characteristics and transmissibility of different types, thereby providing a stronger scientific basis for targeted public health interventions and vaccine development.

## 5. Conclusions

This study analyzed the trends in HPIV-1, HPIV-2, and HPIV-3 incidence by year, season, and age group. The incidence and seasonality differed by type, but the incidence was highest in children for all three types, highlighting the vulnerability of this age group. Once effective HPIV vaccines become available, pediatric immunization should be prioritized as a public health strategy to mitigate the high burden observed in children. In the absence of a vaccine, reinforcing hygiene practices and infection control in childcare facilities, kindergartens, and pediatric wards is essential to reduce transmission. In addition, the development of predictive models to anticipate seasonal epidemics and the implementation of ongoing molecular surveillance are required to strengthen preparedness. This study provides evidence to guide policy and provides a basis for establishing comprehensive national strategies for HPIV prevention and control.

## Figures and Tables

**Table 1:** Distribution of the study participants by age, group, and sex.

Age Group	Male (n) (N = 13,961)	Female (n) (N = 9323)	Total (n, %) (N = 23,284)
Infants (0 years)	2704	1839	4543 (19.5)
Children (1–12 years)	5992	4545	10,537 (45.2)
Adolescents (13–18 years)	336	241	577 (2.4)
Adults (19–64 years)	1893	1042	2935 (12.6)
Older adults (≥65 years)	3036	1656	4692 (20.1)

**Table 2:** Age distribution of HPIV-1, HPIV-2, and HPIV-3 infection by age group.

Age Group	Total No. of Samples (N = 23,284)	HPIV-1 n (%) ^a^	HPIV-2 n (%) ^a^	HPIV-3 n (%) ^a^
Infants (0 years)	4543	88 (1.9)	18 (0.4)	231 (5.0)
Children (1–12 years)	10,537	288 (2.7)	112 (1.0)	528 (5.0)
Adolescents (13–18 years)	577	1 (0.1)	2 (0.3)	19 (3.2)
Adults (19–64 years)	2935	10 (0.3)	3 (0.1)	43 (1.4)
Older adults (≥65 years)	4692	22 (0.4)	6 (0.1)	87 (1.8)

HPIV, Human parainfluenza virus. ^a^ The percentages are row percentages. The denominator for the percentages is all samples tested in the specific age group.

## Data Availability

The data supporting this study originate from patient records at Dankook University Hospital and are protected under ethical and legal regulations. For reasons of privacy and confidentiality, the original datasets are not publicly accessible. Nevertheless, de-identified summary data can be provided by the corresponding author upon reasonable request and with prior approval from the Institutional Review Board.

## Supplementary Materials

The following supporting information can be downloaded at: https://www.mdpi.com/article/10.3390/pathogens14111159/s1, Table S1: Number of positive samples and positivity rate (%) for HPIV types 1, 2, and 3 by year (2007–2024); Table S2: Number of positive samples and positivity rate (%) for HPIV types 1, 2, and 3 by season (2007–2024).

## Abbreviations

The following abbreviations are used in this manuscript:

SDG	Sustainable Development Goals
KINRESS	Korea Influenza and Respiratory Viruses Surveillance System
HPIV	Human parainfluenza virus
PCR	Polymerase chain reaction
RSV	Respiratory syncytial virus
RT-PCR	Real-time reverse-transcription polymerase chain reaction

## References

[1] Branche A.R., Falsey A.R. (2016). Parainfluenza virus infection. Semin. Respir. Crit. Care Med..

[2] DeGroote N.P., Haynes A.K., Taylor C., Killerby M.E., Dahl R.M., Mustaquim D., Gerber S.I., Watson J.T. (2020). Human parainfluenza virus circulation, United States, 2011–2019. J. Clin. Virol..

[3] Shao N., Liu B., Xiao Y., Wang X., Ren L., Dong J., Sun L., Zhu Y., Zhang T., Yang F. (2021). Genetic characteristics of human parainfluenza virus types 1–4 from patients with clinical respiratory tract infection in China. Front. Microbiol..

[4] Howard L.M., Edwards K.M., Zhu Y., Williams D.J., Self W.H., Jain S., Ampofo K., Pavia A.T., Arnold S.R., McCullers J.A. (2020). Parainfluenza virus types 1–3 infections among children and adults hospitalized with community-acquired pneumonia. Clin. Infect. Dis..

[5] Fathima S., Simmonds K., Invik J., Scott A.N., Drews S. (2016). Use of laboratory and administrative data to understand the potential impact of human parainfluenza virus 4 on cases of bronchiolitis, croup, and pneumonia in Alberta, Canada. BMC Infect. Dis..

[6] Liu W.K., Liu Q., Chen D.H., Liang H.X., Chen X.K., Huang W.B., Qin S., Yang Z.F., Zhou R. (2013). Epidemiology and clinical presentation of the four human parainfluenza virus types. BMC Infect. Dis..

[7] Hall C.B. (2001). Respiratory syncytial virus and parainfluenza virus. N. Engl. J. Med..

[8] Wang X., Li Y., Deloria-Knoll M., Madhi S.A., Cohen C., Arguelles V.L., Basnet S., Bassat Q., Brooks W.A., Echavarria M. (2021). Respiratory Virus Global Epidemiology Network. Global burden of acute lower respiratory infection associated with human parainfluenza virus in children younger than 5 years for 2018: A systematic review and meta-analysis. Lancet Glob. Health.

[9] Song Y., Gong Y.N., Chen K.F., Smith D.K., Zaraket H., Bialasiewicz S., Tozer S., Chan P.K., Koay E.S., Lee H.K. (2025). INSPIRE consortium. Global epidemiology, seasonality and climatic drivers of the four human parainfluenza virus types. J. Infect..

[10] Schmidt A.C., Schaap-Nutt A., Bartlett E.J., Schomacker H., Boonyaratanakornkit J., Karron R.A., Collins P.L. (2011). Progress in the development of human parainfluenza virus vaccines. Expert Rev. Respir. Med..

[11] Chen J., Deng S., Xu X., Chen S., Abo Y.N., Bassat Q., Deng J., Komissarov A.B., Liu E., Muñoz-Almagro C. (2025). Regional and type-specific variations in the global seasonality of human parainfluenza viruses and the influence of climatic factors: A systematic review and meta-analysis. The Lancet Glob. Health.

[12] Joung Y.H., Jang T.S., Kim J.K. (2022). Association among sentinel surveillance, meteorological factors, and infectious disease in Gwangju, Korea. Environ. Sci. Pollut. Res. Int..

[13] Laird T.S., Hamilton M., William N., Karanwal S., Marsh K., Evans J. (2025). Trends in human parainfluenza virus in Scotland before and after the peak of the COVID-19 pandemic, January 2017 to October 2023. Euro. Surveill..

[14] Kim H.M., Rhee J.E., Lee N.J., Woo S.H., Park A.K., Lee J., Yoo C.K., Kim E.J. (2022). Recent increase in the detection of human parainfluenza virus during the coronavirus disease-2019 pandemic in the Republic of Korea. Virol. J..

[15] Xu X., Zhang Y., Xu L., Jiang W., Hao C. (2024). Analysis of respiratory pathogen detection in hospitalized children with acute respiratory tract infections after ending the zero COVID policy. Sci Rep..

[16] Park S., Michelow I.C., Choe Y.J. (2021). Shifting patterns of respiratory virus activity following social distancing measures for coronavirus disease 2019 in South Korea. J. Infect. Dis..

[17] Pan L., Yuan Y., Cui Q., Zhang X., Huo Y., Liu Q., Zou W., Zhao B., Hao L. (2024). Impact of the COVID-19 pandemic on the prevalence of respiratory viral pathogens in patients with acute respiratory infection in Shanghai, China. Front. Public Health.

[18] Uyeki T.M., Hui D.S., Zambon M., Wentworth D.E., Monto A.S. (2022). Influenza. Lancet.

[19] Fry A.M., Curns A.T., Harbour K., Hutwagner L., Holman R.C., Anderson L.J. (2006). Seasonal trends of human parainfluenza viral infections: United States, 1990–2004. Clin. Infect. Dis..

[20] Counihan M.E., Shay D.K., Holman R.C., Lowther S.A., Anderson L.J. (2001). Human parainfluenza virus-associated hospitalizations among children less than five years of age in the United States. Pediatr. Infect. Dis. J..

[21] Zhu Z., Zhang Y., Mao N.Y. (2025). Human parainfluenza virus: An important but overlooked respiratory pathogen. World J. Pediatr..

[22] Henrickson K.J. (2003). Parainfluenza viruses. Clin. Microbiol. Rev..

[23] Outlaw V.K., Cheloha R.W., Jurgens E.M., Bovier F.T., Zhu Y., Kreitler D.F., Harder O., Niewiesk S., Porotto M., Gellman S.H. (2021). Engineering protease-resistant peptides to inhibit human parainfluenza viral respiratory infection. J. Am. Chem. Soc..

[24] Rossey I., Saelens X. (2021). Vaccines against human respiratory syncytial virus in clinical trials, where are we now?. Expert Rev. Vaccines.

[25] Scaggs Huang F., Bernstein D.I., Slobod K.S., Portner A., Takimoto T., Russell C.J., Meagher M., Jones B.G., Sealy R.E., Coleclough C. (2021). Safety and immunogenicity of an intranasal Sendai virus-based vaccine for human parainfluenza virus type I and respiratory syncytial virus (SeVRSV) in adults. Hum. Vaccin. Immunother..

[26] Karron R.A., San Mateo J., Thumar B., Schaap-Nutt A., Buchholz U.J., Schmidt A.C., Bartlett E.J., Murphy B.R., Collins P.L. (2015). Evaluation of a live-attenuated human parainfluenza type 1 vaccine in adults and children. J. Pediatr. Infect. Dis. Soc..

[27] Karron R.A., Herbert K., Wanionek K., Schmidt A.C., Schaap-Nutt A., Collins P.L., Buchholz U.J. (2023). Evaluation of a live-attenuated human parainfluenza virus type 2 vaccine in adults and children. J. Pediatr. Infect. Dis. Soc..

