# Supplementary material for: Long-Term Trends in Human Parainfluenza Virus Types 1, 2, and 3 Infection in Korea (2007–2024)
Source: Pathogens. 2025 Nov 14;14(11):1159. doi: 10.3390/pathogens14111159 (PMC12655555; doi:10.3390/pathogens14111159)
Supplement: Supplementary file 1 [file pathogens-14-01159-s001.zip › pathogens-3917143-supplementary.pdf]

Long-Term Trends in Human Parainfluenza Virus Types 1, 2, and 3 Infection in Korea (2007–2024)

Kim *et al.*

Supplementary tables

Table S1. Number of positive samples and positivity rate (%) for HPIV types 1, 2, and 3 by year (2007–2024).

Year	No. of samples tested (N= 23,284)	HPIV-1 n (%)[a] (N = 481)	HPIV-2 n (%)[a] (N = 164)	HPIV-3 n (%)[a] (N = 1,102)
2007	1,057	40 (3.7)	27 (2.5)	75 (7.0)
2008	1,504	68 (4.5)	4 (0.2)	68 (4.5)
2009	1,265	24 (1.8)	18 (1.4)	66 (5.2)
2010	1,657	67 (4.0)	9 (0.5)	71 (4.2)
2011	1,568	43 (2.7)	17 (1.0)	89 (5.6)
2012	1,345	36 (2.6)	1 (0.1)	92 (6.8)
2013	1,545	12 (0.7)	18 (1.1)	64 (4.1)
2014	1,674	48 (2.8)	0 (0.0)	59 (3.5)
2015	1,388	18 (1.2)	14 (1.0)	69 (4.9)
2016	1,645	38 (2.3)	6 (0.3)	98 (5.9)
2017	1,436	18 (1.2)	20 (1.3)	75 (5.2)
2018	1,834	34 (1.8)	5 (0.2)	66 (3.5)
2019	1,432	18 (1.2)	16 (1.1)	67 (4.6)
2020	792	2 (0.2)	6 (0.7)	2 (0.2)
2021	613	0 (0.0)	0 (0.0)	54 (8.8)
2022	860	8 (0.9)	0 (0.0)	13 (1.5)
2023	1,016	4 (0.3)	3 (0.2)	53 (5.2)
2024	653	3 (0.4)	0 (0.0)	21 (3.2)

HPIV, human parainfluenza virus.
[a]The percentages are row percentages. The denominator for the percentages is all samples tested during the specific year.

Table S2. Number of positive samples and positivity rate (%) for HPIV types 1, 2, and 3 by season (2007–2024).

Season	No. of samples tested (N= 23,284)	HPIV-1 n (%)[a] (N = 481)	HPIV-2 n (%)[a] (N = 164)	HPIV-3 n (%)[a] (N = 1,102)
Spring (March to May)	6,391	112 (1.7)	17 (0.2)	534 (8.3)
Summer (June to August)	4,810	148 (3.0)	40 (0.8)	437 (9.0)
Autumn (September to November)	5,607	156 (2.7)	78 (1.3)	99 (1.7)
Winter (December to February)	6,476	65 (1.0)	29 (0.4)	32 (0.4)

HPIV, human parainfluenza virus.
[a]The percentages are row percentages. The denominator for the percentages is all samples tested during the specific season.
